# Evaluating whole transcriptome amplification for gene profiling experiments using RNA-Seq

**DOI:** 10.1186/s12896-015-0155-7

**Published:** 2015-07-30

**Authors:** Sheena L Faherty, C Ryan Campbell, Peter A Larsen, Anne D Yoder

**Affiliations:** Department of Biology, Duke University, Durham, NC 27708 USA

**Keywords:** RNA-Seq, Whole transcriptome amplification, White adipose tissue, Rattus norvegicus

## Abstract

**Background:**

RNA-Seq has enabled high-throughput gene expression profiling to provide insight into the functional link between genotype and phenotype. Low quantities of starting RNA can be a severe hindrance for studies that aim to utilize RNA-Seq. To mitigate this bottleneck, whole transcriptome amplification (WTA) technologies have been developed to generate sufficient sequencing targets from minute amounts of RNA. Successful WTA requires accurate replication of transcript abundance without the loss or distortion of specific mRNAs. Here, we test the efficacy of NuGEN’s Ovation RNA-Seq V2 system, which uses linear isothermal amplification with a unique chimeric primer for amplification, using white adipose tissue from standard laboratory rats (*Rattus norvegicus*). Our goal was to investigate potential biological artifacts introduced through WTA approaches by establishing comparisons between matched raw and amplified RNA libraries derived from biological replicates.

**Results:**

We found that 93% of expressed genes were identical between all unamplified versus matched amplified comparisons, also finding that gene density is similar across all comparisons. Our sequencing experiment and downstream bioinformatic analyses using the Tuxedo analysis pipeline resulted in the assembly of 25,543 high-quality transcripts. Libraries constructed from raw RNA and WTA samples averaged 15,298 and 15,253 expressed genes, respectively. Although significant differentially expressed genes (P < 0.05) were identified in all matched samples, each of these represents less than 0.15% of all shared genes for each comparison.

**Conclusions:**

Transcriptome amplification is efficient at maintaining relative transcript frequencies with no significant bias when using this NuGEN linear isothermal amplification kit under ideal laboratory conditions as presented in this study. This methodology has broad applications, from clinical and diagnostic, to field-based studies when sample acquisition, or sample preservation, methods prove challenging.

**Electronic supplementary material:**

The online version of this article (doi:10.1186/s12896-015-0155-7) contains supplementary material, which is available to authorized users.

## Background

RNA-Seq is quickly becoming the preferred method for comprehensively characterizing global transcriptome activity. This approach has emerged as a powerful tool for determining the link between genotype and phenotype given that the transcriptomes of specific tissue types and individual cells reflect functionality [1-4]. Monitoring changes in gene expression across thousands of genes simultaneously provides an untargeted view of the molecular workings that contribute to adaptive responses to extrinsic and intrinsic stimuli [5,6]. When conducting RNA-Seq studies, one potential obstacle encountered by researchers can result from suboptimal starting quantities of RNA that the use of NGS platforms often requires. This can be especially problematic for certain tissue types wherein starting RNA is in low quantities, such a white adipose tissue, or when using minimally invasive techniques to acquire samples [7-10]. Many of these approaches yield considerably less than a microgram of total RNA [11-16] though current library preparation protocols are typically optimized for a minimum of one microgram of total RNA.

Technologies are emerging to circumvent these challenges. With whole transcriptome amplification (WTA), RNA can be synthetically enriched for gene profiling experiments with both research and diagnostic applications [12,17-19]. WTA amplifies the transcriptome, even in the face of low starting material and/or when samples are heavily degraded due to insufficient preservation [5]. The development and refinement of technologies for high fidelity RNA amplification, therefore, have tremendous appeal for numerous applications spanning the disciplines of field ecology to biomedical diagnostics [12,17,18].

An obvious concern when using WTA for sensitive RNA-Seq is that the replication of transcript abundance occurs without the loss or distortion of transcript expression. Using technologies such as quantitative polymerase chain reaction (qPCR) or microarrays to measure expression levels investigators have compared expression profiles from i.) amplification of varying amounts of starting total RNA [5,20,21]; ii.) amplified and unamplified RNA from the same source [5,14,22,23]; iii.) different commercially available kits or lab-based techniques [20,21,24-26]. These studies are valuable proxies for assessing amplification bias, but these technologies can be limited in their detection capabilities [12,14]. NGS approaches have greater resolution over qPCR and microarrays, and can detect changes in gene expression on a finer scale with digital precision in which one digital unit represents a single mapped sequence read [3,27]. It is therefore critical to assess the accuracy of transcript amplification with the highest resolution technology, particularly when investigators are choosing NGS over other techniques in current experimental methodologies.

Commercially available kits are becoming increasingly prevalent for seamless incorporation of amplified RNA into the NGS pipeline. These are offered by companies such as Clontech, Sigma, Miltenyi Biotec, and NuGEN, and use a variety of techniques to amplify RNA. When considering WTA for RNA-Seq studies, it is important to carefully consider the differences in chemistry and approaches that each commercial kit utilizes. Variations in priming strategies, cDNA synthesis, and amplification of newly converted cDNA can have impacts on length of cDNA products, 3′ bias, amplification efficiency, and fidelity of maintaining relative transcript abundance [1]. All of these factors can potentially introduce unwanted bias in expression studies using RNA-Seq. A researcher limited to using samples collected from the field or from heavily degraded clinical specimens, for example, might consider choosing a kit that uses both oligo (dT) and random hexamers primers for reverse transcription. This might alleviate the potential mis-amplification due to the loss of the poly-A tail from RNA degradation. NuGEN’s Ovation RNA-Seq V2 kit is based on the linear isothermal amplification of double-stranded cDNA that encompasses a unique RNA/DNA heteroduplex at one end using the RNA-dependent DNA polymerase activity [5]. Amplification is initiated at the 3′ end as well as randomly throughout the whole transcriptome in the sample, using both oligoDT and random hexamer primers. This approach will putatively yield a uniform and accurate representation of the transcriptome, an assumption that we directly test in this study.

As commercially available WTA kits continue to improve, investigators should continually reassess their efficiency and compatibility with new sequencing platforms, to determine the best practices as methods evolve. In previous work, Tariq et al. (2011) performed an exploration of the efficacy of the NuGEN Ovation RNA-Seq system by using two different library preparation protocols to reduce ribosomal RNA contamination (i.e. poly-A enrichment and rRNA depletion), and ran their sequencing experiment on two separate sequencing platforms, the SOLiD platform and the Illumina Genome Analyzer IIX, to assess sequencing fidelity and platform-specific biases. Notably, this study attempted to identify the best combination of approaches (i.e. which combination of library preparation protocols and sequencing platforms) will result in the largest amount of high quality data generated from a WTA sample for RNA-Seq characterization. As comprehensive as this investigation was, the authors neglected to determine how WTA technologies, such as the Ovation kit, influence gene expression profiles. Their investigation was instead limited to evaluating differential gene expression between the two sequencing platforms. In addition, since the publication of that study, NuGEN has released an enhanced version of their Ovation kit and Illumina sequencing technology has become more advanced with the advent of their HiSeq platform. In particular, the HiSeq boasts lower error rates, better precision, and longer reads than the GAIIX [7]. Moreover, Shanker et al. (2015) recently conducted a broad-scale investigation of the reproducibility of four commercially-available kits, including NuGEN’s Ovation RNA-Seq V2 system, at multiple laboratories using three varying amounts of starting concentrations on the Illumina HiSeq platform. They find that NuGEN performs remarkably well across multiple laboratories and across technical replicates as low as 500 pg [28]. With RNA-Seq data, however, the largest variability is known to come from biological stochasticity rather than from technical inaccuracies [29,30].

It remains unknown whether WTA preferentially amplifies specific gene products to the exclusion of others. Therefore, our study integrated the design of matched amplified and unamplified RNA from six biological replicates to empirically test this question using NGS transcriptomic sequencing on the Illumina HiSeq 2000 platform, which has become the gold standard for next-generation sequencing experiments [11,13,15,16]. In the present study, we aim to investigate the degree to which NuGEN’s Ovation RNA-Seq V2 system influences gene expression profiles in white adipose tissue excised from standard laboratory rats (*Rattus norvegicus*), building upon and enhancing knowledge regarding current WTA methodologies. To that end, we used the Tuxedo analysis pipeline [2-4,31] to assemble our RNA-Seq reads using the *R. norvegicus* genome, in addition to providing annotation and an exploration of differentially expressed genes. Our study was designed to sequence samples at the greatest depth possible, given the resources at hand, in order to identify any subtle differences between raw RNA and amplified RNA in biological replicates. Ultimately, our goal is to provide foundational insight for future studies to build upon regarding the utility of WTA for clinical and field-based studies that are investigating the functional link between genotype and phenotype via gene expression and RNA-Seq.

## Results

We sequenced over 829 million paired-end reads, 2 ×100 base pairs in length (Lane 1 = 368,942,612, Lane 2 = 460,712,194 reads). Quality filtering resulted in the retention of approximately 90% and 86% of reads from Lanes 1 and 2, respectively (Table 1). Approximately 62% of all reads successfully mapped to the *R. norvegicus* genome (Table 1; Additional file 1: Table S1). Transcript assembly using Cufflinks resulted in 25,543 assembled transcripts.

**Table 1:** **Summary of sequencing results, quality filtering, and transcript assembly from pooled raw RNA samples vs. pooled WTA samples**

	**Raw RNA**	**WTA**
**Total sequenced reads**	368,942,612	460,712,194
**Filtered reads**	334,297,804	395,807,422
**Aligned reads**	208,496,473	241,691,534
**Uniquely aligned reads**	173,011,711	221,886,287
**Multiple aligned reads**	35,484,762	19,805,247
**Unmapped reads**	121,884,321	152,473,973
**Avg. expressed genes**	15,298	15,253

**Figure 1:**
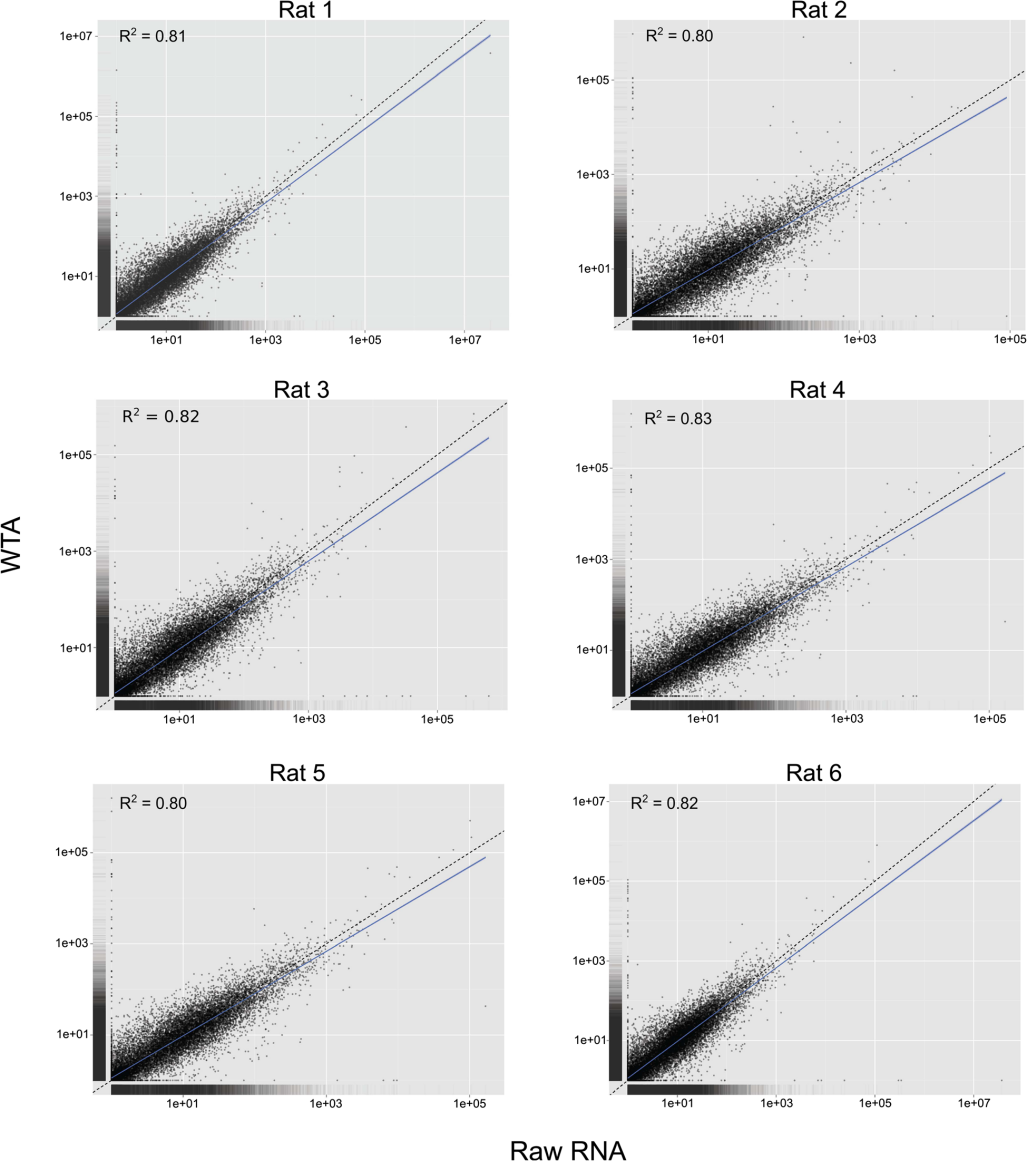
Gene expression scatterplots. FPKM values for all transcripts were plotted, with each dot representing a single transcript. Solid blue lines show the best fit of the data and the dashed line identifies equal expression levels across both conditions.

**Figure 2:**
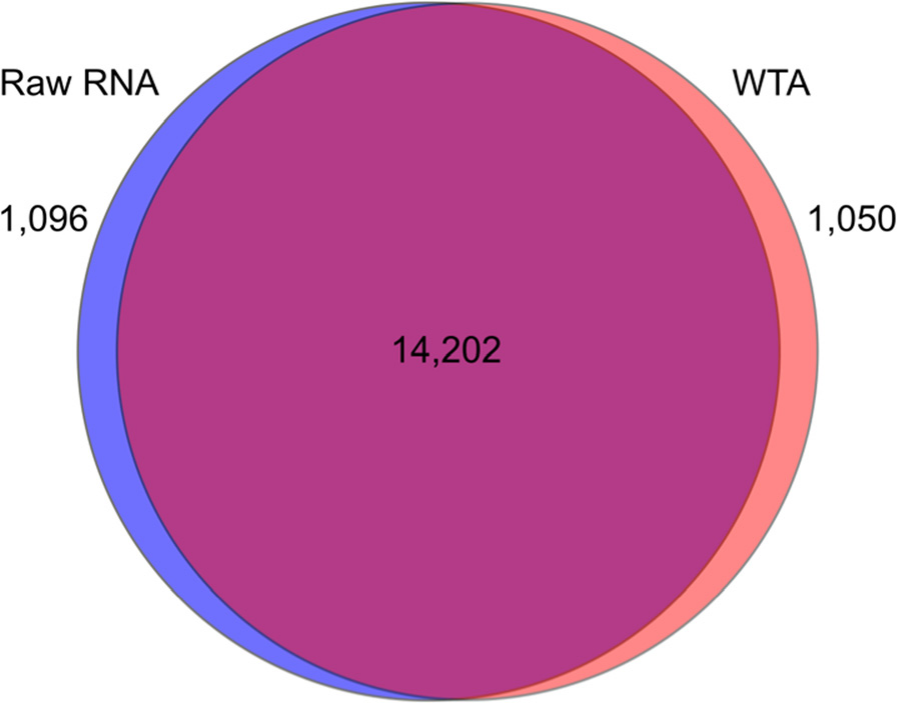
Venn diagram showing overlap among expressed genes. Expressed genes are identified within raw RNA and WTA libraries from all six *R. norvegicus* samples.

**Figure 3:**
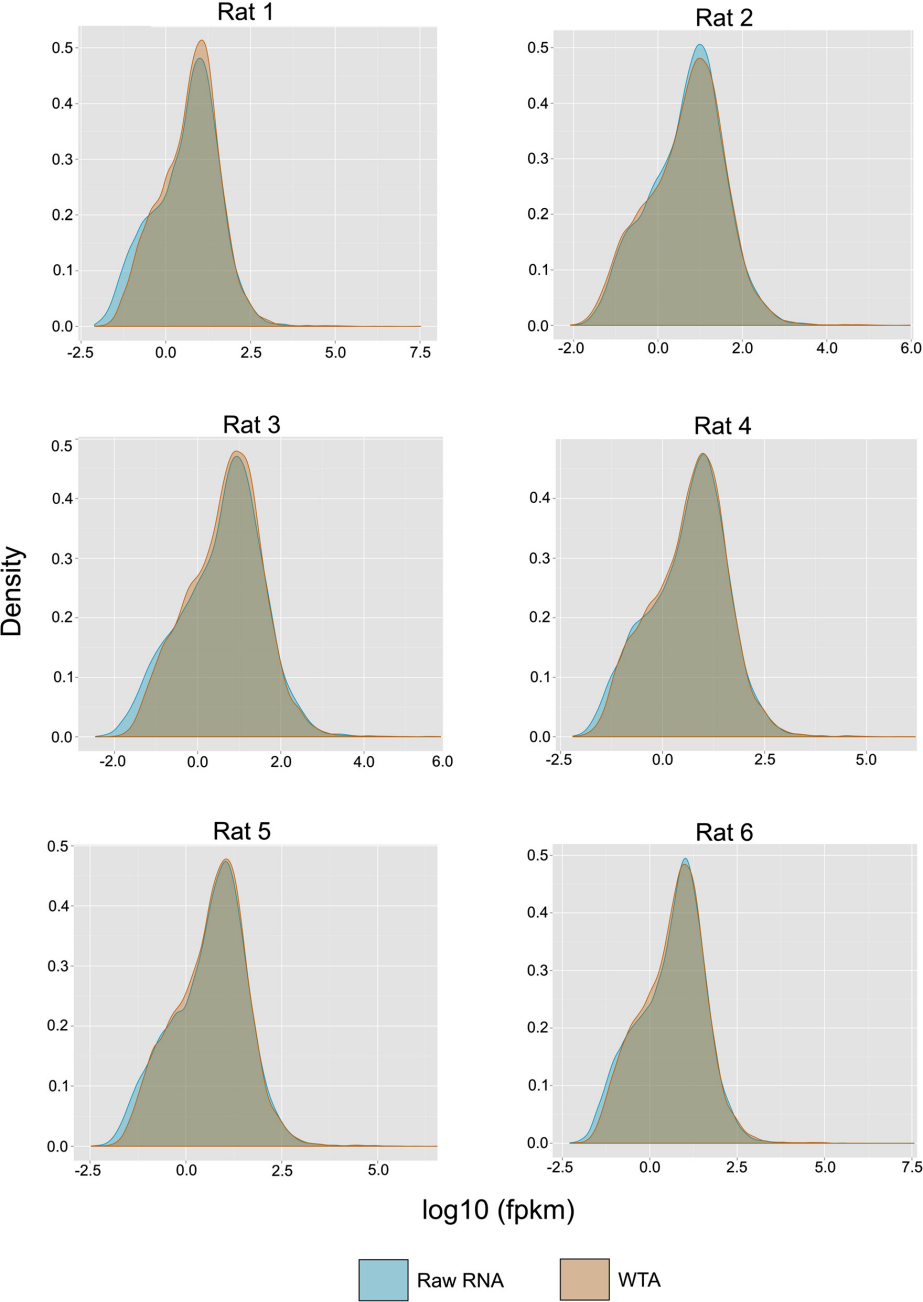
Distribution of gene expression levels across each sample/condition. Comparative distribution analysis of reads correlated to gene density in rat white adipose tissue.

We identified strong correlations in gene expression within each of the six pairwise comparisons of matched raw RNA and amplified RNA, and FPKM (fragments per kilobase of exon per million fragments mapped) values averaged slightly higher in libraries constructed from raw RNA samples (Figure 1). Transcripts with FPKM values greater than 0.05 were identified as being expressed (i.e. above the expected false discovery rate following Trapnell *et al*. 2010), and this threshold was used to determine the number of expressed genes for each sample. Libraries constructed from raw RNA and WTA samples averaged 15,298 and 15,253 expressed genes, respectively (Table 1; Additional file 1: Table S1). Approximately 93% of expressed genes were identical between each of the six matched raw RNA and WTA comparisons (Table 1, Figure 2) and gene density was similar across all samples (Figure 3). Using Cuffdiff, significant differentially expressed genes (P < 0.05) were identified in four of the six comparisons (Table 2), with each of these representing less than 0.15% of all shared genes for each comparison. This percentage is representative of 54 genes, in total, that show differences in expression levels when comparing unamplified vs. amplified. Of the 54 differentially expressed genes, 19 are identified as putative in the Ensembl database as pseudo-genes, while 35 represent functional protein-coding genes, and of those that are protein-coding, 33% percent have homology to human, while the others are putatively rat-specific.

**Table 2:** **Number of significant differentially expressed genes (P < 0.05) per individual identified using Cuffdiff and DESeq**

	**Cuffdiff**		**DESeq**	
	**Differentially expressed genes (Raw RNA vs. WTA)**	**Percent of total shared genes between conditions**	**Differentially expressed genes (Raw RNA vs. WTA)**	**Percent of total shared genes between conditions**
**Rat 1**	0	-	20	0.11%
**Rat 2**	16	0.11%	15	0.08%
**Rat 3**	19	0.14%	23	0.13%
**Rat 4**	18	0.13%	22	0.12%
**Rat 5**	0	-	22	0.12%
**Rat 6**	17	0.12%	25	0.14%

As a complimentary bioinformatic approach to investigate potential differential gene expression, we used HT-Seq [32] to quantify reads by gene, and DESeq [33] to compare these quantities across biological replicates. We find less than 0.14% of genes expressed display differential expression when comparing raw vs. amplified RNA from biological replicates (Table 2). This percentage is representative of 31 genes that show differential expression between unamplified and amplified matched pairs.

**Figure 4:**
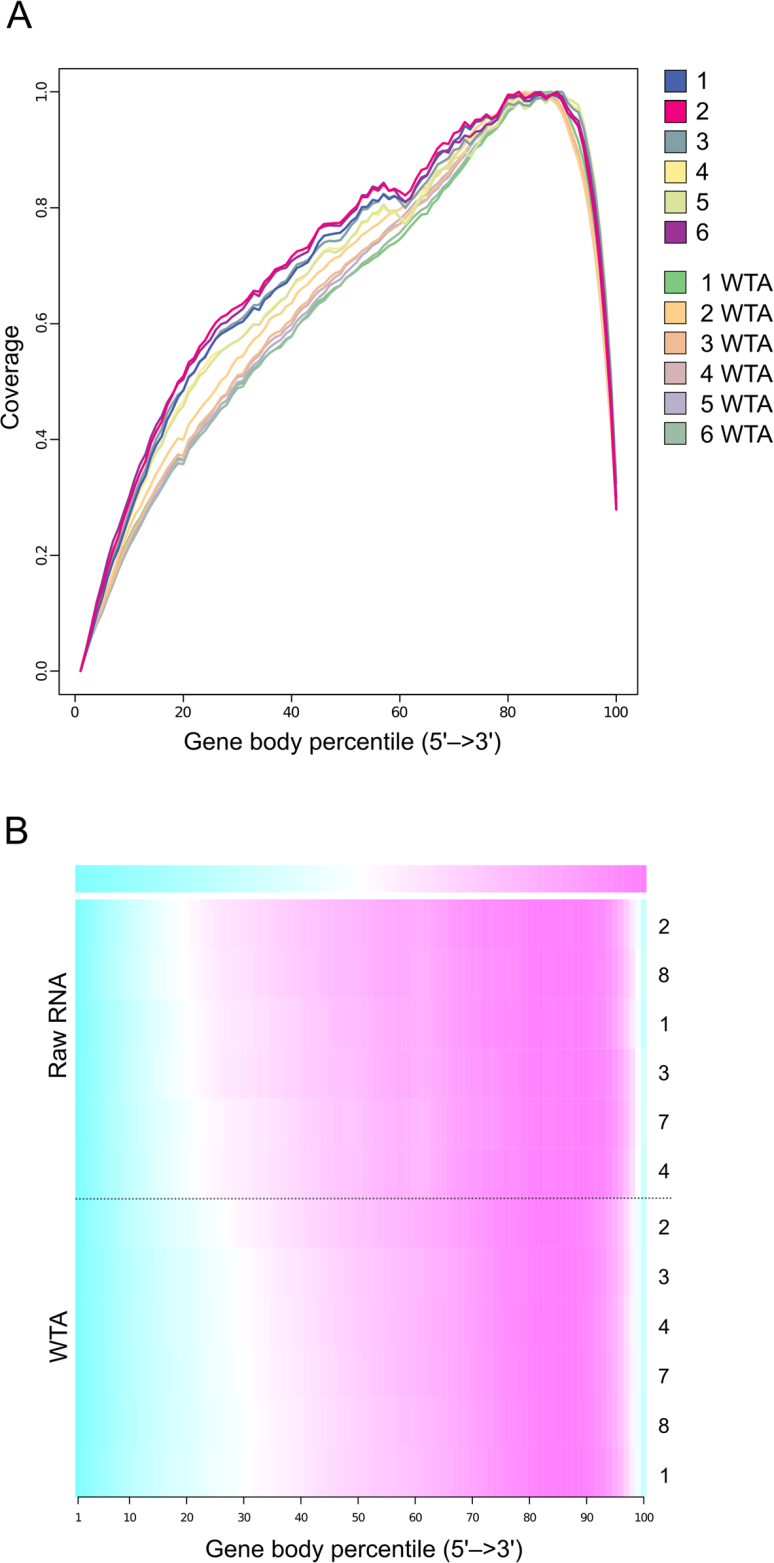
RNA-Seq read coverage of rat gene models. **(A)** Coverage across all transcripts based on mapping of transcriptome reads to the *Rattus norvegicus* genome. All samples showed similar 3′ bias. **(B)** Heat map showing read coverage across all rat genes. Samples are ranked according to Pearson’s skewness coefficients.

We next assessed potential biases in transcript representation (i.e. coverage at 3′ and 5′ end) by determining the average coverage at each percentile of length from 3′ to 5′ end of the known transcripts (Figure 4). The coverage depth analysis at the extreme 3′ and 5′ ends of the transcripts confirms 3′ bias for all of our samples independent of WTA treatment and is likely an artifact of library creation, which were prepared using a poly-A-based enrichment method.

## Discussion

RNA-Seq has enabled the sequencing of transcriptomes to allow both identification and quantification of transcript levels. The digital precision and sensitivity of RNA-Seq is well-suited to the analysis of low-input or degraded samples, especially with respect to older methods such as qPCR or microarrays, yet many available protocols require total RNA around 1.0 microgram, presenting a potential obstacle for clinical or field-based studies. Here, we have tested the efficacy of NuGEN’s Ovation RNA-Seq V2 system for whole transcriptome amplification by comparing matched amplified and unamplified samples to identify if there exists any subtle changes in gene expression after WTA among biological replicates. We were specifically interested in investigating the degree to which accurate representations of gene diversity and transcript abundance levels were achieved when sequenced on an Illumina HiSeq.

### Gene expression patterns in unamplified versus amplified libraries

We identified consistent patterns of gene presence and absence across each of the six comparisons, with an average overlap of approximately 93%. We found no evidence that one or more specific gene products were differentially expressed in matched unamplified vs. amplified RNA in this study. Using Cuffdiff, the only gene found to be differentially expressed in as many as four of the six sample pairs was classified as a pseudo-gene and one protein-coding gene, heterogeneous nuclear ribonucleoprotein A0 (*Hnrnpa0*), a DNA binding protein that is thought to influence pre-mRNA processing, mRNA metabolism and transport, was found to be differentially expressed in three out of the six sample pairs. The remaining differentially expressed genes were only found in two or fewer comparisons, and no pairwise comparison shared more than five genes. Our results from DESeq also indicate that there is no consistent biological distortion, as 48% (15 out of 31) of differentially expressed genes were found in five or fewer of our pairwise comparisons. This result is remarkably consistent with the differential expression results produced using Cuffdiff. Moreover, we observe no overlap with the genes that Cuffdiff identified as differentially expressed, indicative of the small proportion of differentially expressed genes being a product of background noise from stochastic events observed with typical RNA-Seq experiments. This result thus lends strong support to our hypothesis that there is no consistent biological distortion when using WTA.

### Testing the efficacy of NuGEN’s Ovation RNA-Seq V2 kit

Only a few studies have thus far investigated potential biases in gene expression introduced by WTA approaches using NuGEN’s WTA technology [5,21,28,34]. These studies have been foundational in determining that the WTA chemistry tested herein performs well across technical replicates of varying amounts of starting material down to the limits of 50–100 pg. However, to our knowledge, no previous work has included biological replicates in their analyses. Thus, the strength of our investigation is that it incorporates the experimental design of matched amplified and unamplified RNA from six biological replicates to test for preferential amplification of WTA approaches. In the present study, we do not find that WTA selectively amplifies specific gene products and the small variability observed among replicates is most likely attributed to background noise. Biological replicates are essential for identifying potential distortion from gene expression stochasticity inherent in any biological system.

We chose NuGEN’s Ovation kit based upon the single primer isothermal amplification (SPIA) technology for achieving WTA. This technology incorporates a combination of 3’-Ribo-SPIA (i.e. polydT) primers, which prime synthesis of cDNA at the 3’ polyA tail, and whole transcript (WT)-Ribo-SPIA (i.e. random hexamer) primers to prime cDNA synthesis across the full length of the transcripts, providing amplification independent of sample degradation at the 3’ end [5,8-10,35]. Ideally, this feature makes the Ovation RNA-Seq System V2 kit useful for NGS platforms, such as the Illumina HiSeq which function by distributing reads across the entire length of the transcript. Previous studies have also found that commercial kits show high reproducibility in WTA procedures completed by researchers in different laboratories (correlation coefficient of ~0.95), thus providing encouraging support for the use of WTA prior to sequencing when working with challenging samples [12,14,21].

### Utility of whole transcriptome amplification for non-model species or field-based studies

Studies that investigate the relationship between genotype and phenotype have largely focused on model organisms for which a genome is publicly available, and have been performed using captive populations, or with cell culture methods under controlled laboratory settings for which sample material is relatively easy to obtain [12,17,18,36-46]. Although these studies provide a wealth of vital information on the drivers of physiology, behavior, and disease dynamics, comparable studies on non-model organisms or studies that use crucial pathological specimens (e.g. laser capture microdissection, FFPE, or biopsied tissue) or samples collected in the field remain scarce. This is due to the challenging nature of working with these sample types, often adding another layer of complexity (e.g. potential degradation) to the already present challenge of low RNA input [5,12,20,21,47-50]. Potential degradation is due to the fact that RNA is highly sensitive to endogenous and exogenous RN*ases*, which rapidly degrade RNA molecules at ambient conditions [5,14,22,23,51,52]. Degradation can result in immediate changes in expression profiles or a prospective loss of rare transcripts immediately after sample collection [20,21,24-26,53,54]. Although, this issue may be unavoidable for certain experiments, the priming strategy of the NuGEN kit may alleviate potential downstream issues when using partially degraded samples.

## Conclusions

Our study builds upon and enhances existing knowledge regarding the NuGEN Ovation RNA-Seq V2 system’s performance in studies that use WTA for RNA-Seq experiments. Previous studies have demonstrated that this chemistry performs well across technical replicates of varying starting amounts of input RNA and across multiple laboratories. The novelty of our study is that it empirically tests biological variation, by incorporating the design of matched amplified and unamplified RNA from six biological replicates. The Ovation RNA-Seq V2 kit is able to amplify small quantities of RNA from tissue samples while maintaining transcript levels nearly identical to those from matched unamplified input RNA, demonstrating an overlap of 93% when comparing each matched pair individually. Despite this high fidelity of transcript representation, future studies should anticipate the slight discrepancy revealed here and take measures to account for the potential for small differences in downstream analyses. Additionally, future work could benefit from testing this methodology using more realistic scenarios, such as repeating the experiment with smaller input concentrations, or using partially degraded samples that may be more representative of RNA that may be extracted from clinical or from field-based studies. Nonetheless, this study provides the essential first step into investigations into the putative alteration of gene expression profiles due to WTA among biological replicates.

## Methods

### Tissue sampling

Animal handling was carried out in strict accordance with the approval of Duke University’s Institutional Animal Care and Use Committee (IACUC protocol #A096-13-04). Gonadal white adipose tissue was excised from standard laboratory rats (*Rattus norvegicus*, n = 6) and snap frozen in liquid nitrogen. Samples were transferred to −80°C and stored there until sample processing.

### RNA extraction

Approximately 100 mg of white adipose tissue was used for total RNA extraction. RNA was purified using an optimized TRIzol protocol in conjunction with the Microarray Tissue Kit (Qiagen; Valencia, CA, USA). Briefly, frozen tissues were homogenized in 1 ml TRI Reagent (Ambion; Grand Island, NY, USA) using a hand-held rotor-stator homogenizer to provide efficient disruption and homogenization of samples. BCP (1-bromo-3-chloropropane) extraction was performed using 100 ul of BCP, followed by centrifugation at 12,000 × g for 15 min at 4°C. Aqueous layer containing total RNA was transferred to a fresh 1.5 ml centrifuge tube and one volume of 70% EtOH was added to sample. The entire sample volume was loaded onto Qiagen filter columns and kit protocol was followed according to manufacturer’s instructions, including an on-column DN*ase* step to remove residual contaminating DNA before amplification and downstream sequencing.

RNA integrity and concentration was assessed using the 2100 Bioanalyzer (Agilent Technologies; Santa Clara, CA, USA) at the Duke Institute for Genome Science and Policy’s Microarray Facility. RNA integrity is provided by a RNA Integrity Number (RIN), which is calculated based on the comparison of the areas of 18S rRNA and 28 s rRNA [12,14,55]. Values range from 1 being the most degraded to 10 being the most intact. RIN values for our total RNA extractions ranged from 9.2 – 9.4, a measure of high quality (Additional file 2: Figure S1). Total RNA concentrations ranged from 200–300 ng/ul.

### Whole transcriptome amplification

Total RNA extractions were divided into two tubes for each rat, for a total of 12 tubes. Six tubes were normalized to 100 ng/μl in a 5 μl volume, for a total of 500 ng of RNA, and subjected to whole transcriptome amplification using NuGEN’s Ovation RNA-Seq V2 kit (San Carlos, CA, USA) according to manufacturer’s instructions. This kit provides a rapid method for preparing amplified cDNA from total RNA for downstream RNA-Seq applications. It employs a single primer isothermal amplification (SPIA) method to amplify total RNA into double stranded cDNA and depletes rRNA without preselecting mRNA. Amplified cDNA samples were then purified using the MinElute Reaction Cleanup Kit (Qiagen; Valencia, CA, USA) according to manufacturer’s protocol. After the amplification procedure, cDNA concentrations ranged from 421–575 ng/ul (Additional file 1: Table S2). Paired amplified cDNA (n = 6) and unamplified raw RNA samples (n = 6) were then sent to Duke GCB Genome Sequencing Shared Resource for library preparation and sequencing.

### Library preparation and sequencing

Total RNA samples were converted to cDNA libraries using Illumina’s TruSeq RNA Sample Preparation Kit (San Diego, CA, USA). In summary, 1 μg of total RNA was enriched for mRNA using oligo-dT coated magnetic beads, fragmented, and reverse transcribed into cDNA. The cDNA was fragmented into smaller pieces, blunt-ended, and ligated to indexed (barcoded) adaptors and amplified using PCR. Previously amplified cDNA libraries were prepared for sequencing using Illumina’s TruSeq DNA Sample Preparation Kit according to manufacturer’s protocol. Final library size distribution was determined using Agilent Bioanalyzer 2100. Insert size was between 120–130 base pairs (bp) and average library size was 230 bp. Twelve libraries were multiplexed, pooled and sequenced on two lanes of the Illumina HiSeq2000 platform (San Diego, CA, USA) using 100 bp paired-end reads. Specifically, six multiplexed non-amplified samples were sequenced on Lane 1, while six multiplexed amplified samples were sequenced on Lane 2. The data set supporting the results of this article are available in the NCBI Short Read Archive repository [accession number SRP049463; http://www.ncbi.nlm.nih.gov/sra/?term=SRP049463].

### Read mapping and transcriptome assembly

RNA-Seq reads were filtered using the Trimmomatic program [56] and aligned to the *R. norvegicus* genome (Rnor_5.0; downloaded from Ensembl.org on 3/2014) using TopHat2 (v 2.0.5) [3,27,57]. The resulting alignment data from TopHat2 were then fed into Cufflinks (v 2.0.2) to assemble aligned RNA-Seq reads into transcripts for each rat individually. Annotated transcripts were obtained from Ensembl database (Rnor_5.0.75.gtf; downloaded from Ensembl.org on 3/2014). Transcript abundances estimates were measured in FPKM. Cuffdiff was used to determine differential gene expression profiles between amplified and unamplified matched samples for each biological replicate included in our study [58]. HTSeq was used under default parameters to produce gene counts for each of our twelve samples [32]. To estimate differential expression, we fed the matrix of read counts generated by HTSeq into DESeq [33]. Since we were working with biological, and not technical replicates, we reduced the read counts to two conditions for each sample (amplified and raw) and estimated dispersion by ignoring condition and treating all samples as if they were replicates of the same condition. We used the option sharingMode = “fit-only” to retain outliers, accounting for biological replications in our experimental design. The geneBody_coverage.py script within the RseqC package [59] was used to examine read coverage across gene models and test for 5′ and 3′ bias in transcript coverage.

## Additional files

Additional file 1: Table S1. Summary of sequencing results, quality filtering, and transcript assembly from biological replicates included in our analysis. **Table S2.** Summary of pre- and post-amplification RNA concentrations. Additional file 2: Figure S1. Agilent Bioanalyzer traces demonstrate total RNA extractions are high-quality.

## Abbreviations

WTA: Whole transcriptome amplification
qPCR: Quantitative polymerase chain reaction
FPKM: Fragments per kilobase of exon per million fragments mapped
BCP: 1-bromo-3-chloropropane
RIN: RNA integrity number
SPIA: Single primer isothermal amplification
