# Supplementary figures and images for: Evaluating whole transcriptome amplification for gene profiling experiments using RNA-Seq
Source: BMC Biotechnol. 2015 Jul 30;15:65. doi: 10.1186/s12896-015-0155-7 (PMC4520150; doi:10.1186/s12896-015-0155-7)

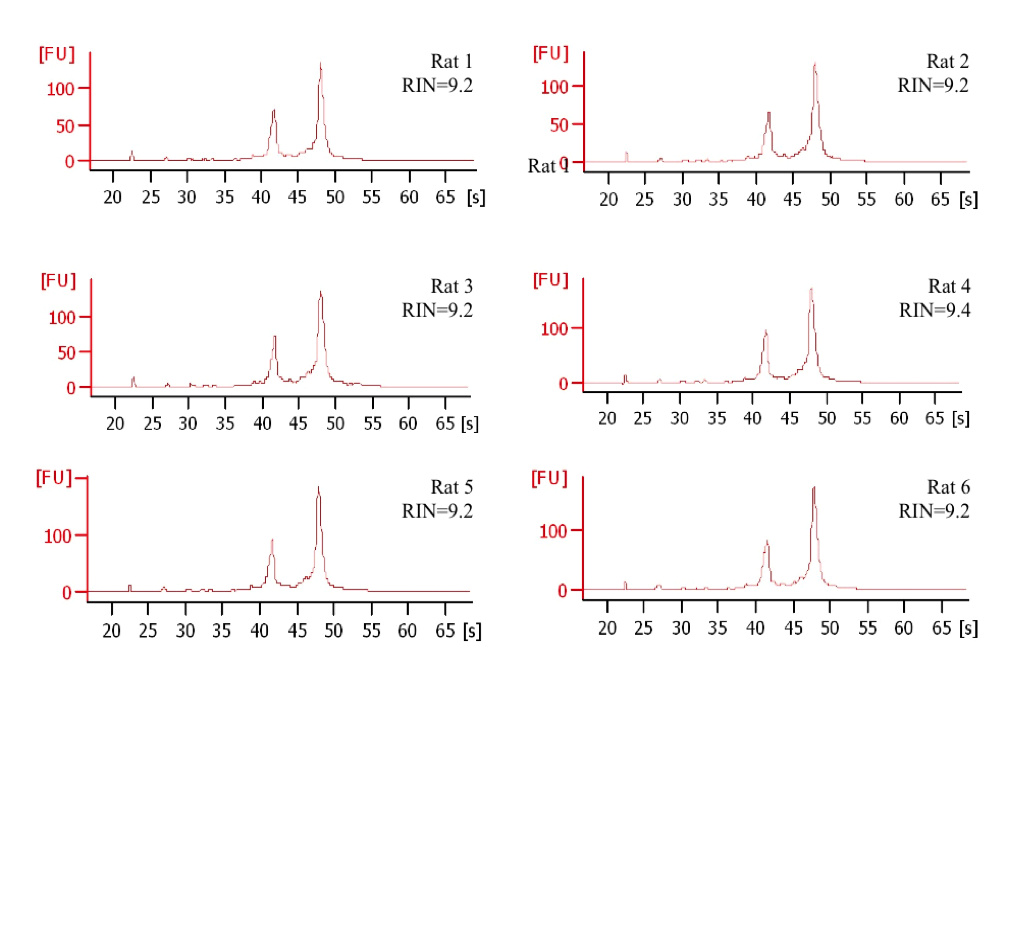

Supplement: Additional file 2: Figure S1. — Agilent Bioanalyzer traces demonstrate total RNA extractions are high-quality. [file 12896_2015_155_MOESM2_ESM.png]
